# Isolation and draft genome sequence of *Paenibacillus* sp*.* CCS19

**DOI:** 10.7150/jgen.87228

**Published:** 2023-09-11

**Authors:** Hironaga Akita, Yoshiki Shinto, Zen-ichiro Kimura

**Affiliations:** 1 College of Industrial Technology, Nihon University, 1-2-1 Izumi-cho, Narashino, Chiba 275-8575, Japan.; 2 Department of Civil and Environmental Engineering, National Institute of Technology, Kure College, 2-2-11 Aga-minami, Kure, Hiroshima 737-8506, Japan.

**Keywords:** *Paenibacillus*, Oligotroph, Enzyme, 16S rRNA, Draft genome sequence

## Abstract

Here, we describe the isolation and draft genome sequence of *Paenibacillus* sp. CCS19. *Paenibacillus* sp. CCS19 was isolated from leaf soil collected in Japan and identified based on similarity of the 16S rRNA sequence with related *Paenibacillus* type strains. The draft genome sequence of *Paenibacillus* sp. CCS19 consisted of a total of 107 contigs containing 6,816,589 bp, with a GC content of 51.5% and comprising 5,935 predicted coding sequences.

## Introduction

The isolation and identification of previously unknown bacteria could lead to the development of new industrial technologies. For example, in a previous study by Akita et al., thermostable NADP^+^-dependent *meso*-diaminopimelate dehydrogenase (*meso*-DAPDH) was newly discovered from *Ureibacillus thermosphaericus*, a bacterium isolated from compost 1. Moreover, NADP^+^-dependent D-amino acid dehydrogenase (D-AADH) was created from *meso*-DAPDH by protein engineering 2. D-Amino acids are important chiral intermediates for agrochemicals, semisynthetic antibiotics, and pharmaceutical drugs, but their utilization is limited by high production costs. D-AADH can be used in one-step production of several D-amino acids by reductive amination of the corresponding 2-oxo acids with ammonia and NADPH in a process involving fewer steps than current industrial-scale production methods 2. Thus, production methods using D-AADH have potential for industrial use because manufacturing costs can be reduced.

Several types of oligotrophic microorganisms exhibit high growth rates under low-nutrient conditions; thus, the metabolic enzymes from oligotrophs may have high catalytic activity. Moreover, as mentioned above, such enzymes are also potentially useful in industrial applications. Recently, using our newly developed method for the isolation and identification of novel oligotrophs, we identified *Deinococcus kurensis* KR-1^T^ 3, *Enterobacter oligotrophicus* CCA6^T^ 4, *Pseudomonas humi* CCA1^T^ 5, and* Paenibacillus glycanilyticus* subsp. *hiroshimensis* CCI5^T^ 6. Here, we report the isolation and identification of another oligotroph, strain CCS19.

## Materials and Methods

Soil samples such as compost, leaf soil, mud, and peat moss were collected at Narashino City in Chiba Prefecture, Japan. Oligotrophs were isolated from the soil samples on 1.5% agar plates (pH 7.2) containing sulfate (<0.4%), calcium (<0.1%), iron (<0.01%), and a few fatty acids and/or other minerals at concentrations of less than 0.01%. Soil samples were suspended separately at 10% (w/v) in sterilized water and then filtered. The filtrates were inoculated separately onto 1.5% agar plates and incubated for 2 days at 37°C. Individual colonies were successively re-streaked onto fresh 1.5% agar plates at least three times to obtain pure colonies, and the pure colonies were used for phylogenetic characterization.

After incubation in R2A broth at 37°C for 2 days, the cultures were washed with sterile water. Subsequently, genomic DNA was extracted using an Illustra^TM^ bacteria genomicPrep Mini Spin Kit (GE Healthcare, Chicago, IL, USA) according to the manufacturer's instructions. The concentration and purity of the extracted genomic DNA were measured using a Quant-iT dsDNA Assay Kit (Invitrogen) and a NanoDrop ND-1000 spectrophotometer (Thermo Fisher Scientific), respectively.

Using the extracted genomic DNA as a template, the 16S rRNA gene was amplified using KOD-plus DNA Polymerase (TOYOBO, Osaka, Japan) with the bacterial universal primers 27f 7 and 1391r 8. After the amplified PCR product was purified using a Wizard SV Gel and PCR Clean-Up System (Promega, Madison, WI, USA), the purified product was cloned into the pTA2 vector (TOYOBO), yielding pTA2/16S, which was then sequenced. The sequence of the 16S rRNA gene (accession number: LC763767; 1512 bp) was compared with reference sequences available in the GenBank/EMBL/DDBJ databases using BLAST. Multiple alignment and construction of a maximum-likelihood tree were performed using MEGA-X 9 with the Tamura-Nei model 10.

Sequence libraries for genome sequencing were prepared using a Nextera XT DNA Library Preparation Kit (Illumina, San Diego, CA, USA) according to the manufacturer's instructions. The resulting libraries were sequenced using a MiSeq sequencer (Illumina) with a MiSeq Reagent Kit v3 (Illumina). Default parameters were used for all software unless otherwise specified. Quality control and* de novo* assembly were carried out using Trimmomatic ver.0.39 11 and Shovill ver.1.1.0 12, respectively. Genome annotation was carried out using DFAST ver.1.2.0. 13.

## Results and Discussion

To isolate the objective oligotrophs, we screened samples on 1.5% agar plates without a carbon source and other medium components, and a few colonies were formed when filtrate prepared from leaf soil was plated. After standard dilution plating was carried out for the purification of colonies, a colony exhibiting rapid growth was obtained and designated strain CCS19.

**Figure 1:**
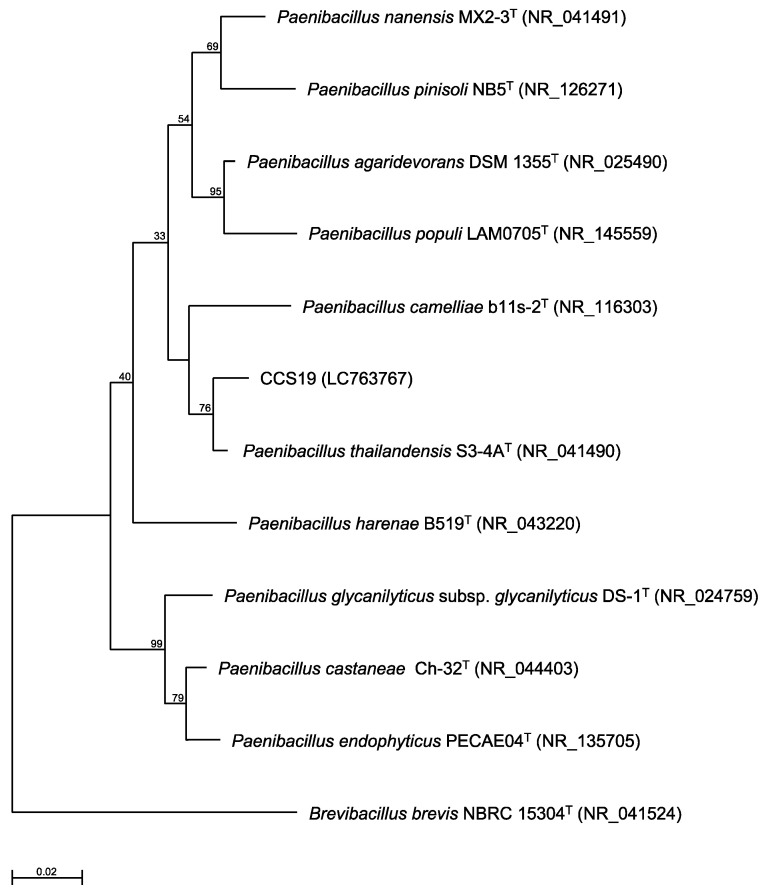
Phylogenetic tree constructed from analysis of 16S rRNA gene sequences and showing the relationships between strain CCS19 and related *Paenibacillus* type strains. The bar indicates a 0.02% nucleotide substitution rate. The tree was rooted using *Brevibacillus brevis* NBRC 15304^T^ as the outgroup.

To identify strain CCS19, the 16S rRNA gene sequence was determined and used to construct a phylogenetic tree. Strain CCS19 clustered with members of the genus* Paenibacillus* (Figure 1). In particular, strain CCS19 exhibited similarities of 98.7%, 98.1%, 97.2%, 96.7%, 96.6%, 96.6%, 96.3%, and 96.3% to its closest relatives, *P. thailandensis* S3-4A^T^, *P. nanensis* MX2-3^T^, *P. agaridevorans* DSM1355^T^, *P. castaneae* Ch-32^T^, *P. harenae* B519^T^, *P. populi* LAM0705^T^, *P. camelliae* b11s-2^T^, and *P. pinisoli* NB5^T^, respectively. Based on these results, we identified strain CCS19 as *Paenibacillus* sp. CCS19.

Most type strains in the genus *Paenibacillus* are characterized as gram-positive or -negative, aerobic or facultative anaerobic, rod-shaped, motile bacteria. Currently, more than 300 species and 6 subspecies of *Paenibacillus* have been identified based on 16S rRNA gene sequence homology as well as physiological and chemotaxonomic characteristics (https://www.bacterio.net/genus/paenibacillus). Moreover, *Paenibacillus* were found to produce novel enzymes exhibiting substrate specificity different from that of known enzymes such as alcohol oxidase 14 and (*2R*,*3R*)-2,3-butanediol dehydrogenase 15. To enable the use of enzymes produced by *Paenibacillus* sp. CCS19, the draft genome sequence was determined. The raw data after genome sequencing using a MiSeq sequencer yielded 64,617 reads with 105-fold coverage. The assembled genome sequence of *Paenibacillus* sp. CCS19 contained 107 contigs, which consisted of 6,816,589 bp and GC content of 51.5%. Moreover, within the draft genome sequence of *Paenibacillus* sp. CCS19, a total of 5,935 predicted coding sequences were identified.

To facilitate the screening and isolation of novel enzymes, in the present study, we isolated and determined the draft genome sequence of *Paenibacillus* sp. CCS19. Analysis of the draft genome sequence of *Paenibacillus* sp. CCS19 could reveal novel enzymes suitable for industrial use.

### Nucleotide Sequence Accession Number

The draft genome sequence of *Paenibacillus* sp. CCS19 was deposited in the DDBJ/EMBL/GenBank databases under accession numbers BTCK01000001 to BTCK01000158. The raw sequence reads were deposited in DDBJ under BioProject number PRJDB15611 and BioSample number SAMD00590491.
